# Salinity Tolerance in Plants: Trends and Perspectives

**DOI:** 10.3390/ijms20102408

**Published:** 2019-05-15

**Authors:** Jose Antonio Hernández

**Affiliations:** Group of Fruit Trees Biotechnology, Dept. Plant Breeding, CEBAS-CSIC, Campus Universitario de Espinardo, 25, 30100 Murcia, Spain; jahernan@cebas.csic.es; Tel.: +34-968-396-200

Salinity stress is one of the more prevailing abiotic stresses which results in significant losses in agricultural crop production, particularly in arid and semi-arid areas. According to FAO, around 800 million hectares of land are affected by salinity worldwide. Therefore, it is of vital importance to know the mechanisms of salinity tolerance in order to obtain plants with a better response to this abiotic stress. At the same time, it is necessary to achieve these objectives with sustainable agricultural practices that allow obtaining more productive crops under a future scenario of climate change.

The first symptoms from the early hours until a few days later associated with saline stress are displayed in the roots by suffering an osmotic stress associated with the accumulation of phytotoxic ions. In the long term, salinity induces ion toxicity due to a nutrient imbalance in the cytosol. In addition, salt stress is also manifested as an oxidative stress at the subcellular level, mediated by reactive oxygen species (ROS) [1]. All these responses to salinity contribute to deleterious effects on plants, although there are tolerant plants to NaCl that can implement a series of adaptations to acclimate to salinity that can help their survival. These adaptation mechanisms include morphological, physiological, biochemical, and molecular changes [1].

The majority of research on salt tolerance in plants in this Special Issue is focused on determining which genes are involved in the molecular mechanisms of tolerance. Likewise, there are also an important number of works using transgenic plants in order to get a better response to salinity.

## 1. Transcriptomic and Genomic Approaches

Transcriptome sequencing may provide a functional view of the plant resistance mechanisms to salt stress. Wang et al. [2] performed a transcriptome analysis of short-term acclimation (for 24 h) in the algae *Chlamydomonas reinhardtii* to salt stress (200 mM NaCl) [2]. The authors identified 10,635 unigenes as differentially expressed in *C. reinhardtii* under salt stress by RNA-seq, including 5920 that were up-regulated and 4715 that were down-regulated. Using GO (gene onthology) terms, MapMan, and KEGG (Kyoto Encyclopedia of Genes and Genomes) functional enrichment analyses, the potential mechanisms for responses to salt stress were identified [2]. These analyses reported that lipid homeostasis and the regulation of phosphatidic acid acetate levels had a key role in improving tolerance to salt stress, and use as an alternative source of energy for solving the impairment of photosynthesis and the enhancement of glycolysis metabolism [2].

By using also *C. reinhardtii* as an experimental organism, [3] evaluated the role of the basic leucine-region zipper (bZIP) transcription factors (TFs) in response to salt stress [3]. They identified, using a genome-wide analysis, 17 *C. reinhardtii* bZIP (*CrebZIP*) TFs containing typical bZIP structure, as well as the CrebZIP gene structures and their chromosomal assignment were also analysed [3]. The expression profiling of *CrebZIP* genes by qRT-PCR indicated that six *CrebZIPs* might be involved in stress response and lipid accumulation. The authors also concluded that CrebZIPs TFs may play important roles in mediating photosynthesis, as suggested by the reported reduced chlorophyll content and Fv/Fm, and the increased NPQ, carotenoid, and oil contents which could be interpreted as adaptive mechanisms to salt stress [3].

Wu et al. [4] sequenced the flax (*Linum usitatissimum* L.) transcriptome to identify differentially-expressed unigenes (DEUs) under NaCl stress [4]. After the results of the flax transcriptome were confirmed using qRT-PCR, a large-scale analysis of expressed sequence tag-derived simple sequence repeat (EST-SSRs) markers was conducted using public resources in order to understand the functions of the identified genes. The authors identified 33,774 significant DEUs (18,040 up-regulated and 15,734 down-regulated) [4]. The functional categories of the DEUs were mostly assigned as signal transduction of plant hormones, photosynthesis-antenna proteins, and biosynthesis of amino acids, which are important in flax responses to NaCl exposure [4]. They also identified a number of DEUs homologous to known plant transcription factors that regulate abiotic stress responses, such as *bZIP*, *HD-ZIP*, *NAC*, *MYB*, *GATA*, *CAMTA,* and *B3*. The authors also suggested an important role of the bZIP TFs in the response to salinity [4].

Grapevine (*Vitis vinifera*) is an economically important fruit crop. This fact makes the search for salt-tolerant genotypes a relevant issue. Recently, next-generation sequencing (NGS) technology based on high throughput RNA-Seq technology has been extensively used to unveil and compare the transcriptome profile under abiotic stresses [5], which provides large-scale data to identify and characterize the differentially-expressed genes DEGs. Guan et al. [6] carried out the transcriptomic sequencing of cDNA generated from both control and salt-treated grapevine leaf samples [6]. The GO and KEGG analyses of DEGs in response to salt stress suggested that many genes were involved in various defense-related biological pathways, including ROS scavenging, ion transport, heat shock proteins (HSPs), pathogenesis-related proteins (PRs), and hormone signaling. Furthermore, many DEGs encoded for TFs and essential regulatory proteins involved in signal transduction by regulating genes associated with salinity in grapevine [6]. The authors also observed that salinity negatively affected all gas exchange parameters. The analysis of antioxidant enzymes showed that at short-term (up to 60 h) salinity significantly increased the superoxide dismutase, peroxidase, catalase, and glutathione S-transferase activities in grapevine leaves, suggesting that salt stress induced an oxidative stress. Regarding the ion contents, they showed that Cl^-^ accumulated in the roots more than in the leaves [6], and this response can be considered as an adaptive mechanism limiting the accumulation of these ions in the canopy [1]. Finally, the authors proposed four genes as candidates as potential markers of salt stress (nonspecific lipid-transfer protein, *LTP*; proline-rich cell wall protein-like, *PRPs*; glutathione transferase-like, *GST*; photosystem II reaction center protein, with the aim of explore new approaches to applying the gene information in genetic engineering and breeding purposes [6].

Yu et al. [7] performed a genome-wide association study (GWAS) of salt-tolerance-related phenotypes in rice during the germination stage, in order to identify candidate genes related to salt tolerance [7]. In that regard, they characterized 17 genes that may contribute to salt tolerance during the seed germination stage, which contained highly associated SNPs (single-nucleotide polymorphisms)/indels whiting the coding region. Among these genes, *OSMADS31* is involved in floral organ specification as well as in plant growth and development. *OSHAK11* coded for a high-affinity K^+^ uptake transporter, significantly induced by salt-stress and K^+^ starvation. *AGOs* play important roles in the regulation of development and stress responses. *OsPIN* is encoded for an auxin efflux carrier protein, and is involved in the root elongation growth and lateral root formation. *Germin family proteins*, is involved in plant cell defense and diseases. Finally, *SAP* (A20/AN1 zinc finger containing proteins) and zinc-induced facilitator-like (*ZIFL*) family genes are involved in the regulation of stress signaling in plants [7].

Using the same approach (GWAS mapping), Dilnur et al. [8] revealed nine SNP-rich regions significantly associated with plant performance parameters [the relative fresh weight (RFW), the relative stem length (RSL), the relative water content (RWC)] and the comprehensive index of salt tolerance (CIST) in 215 accessions of Asiatic cotton (*Gossypium arboretum* L.) grown in the absence or the presence of 150 mM NaCl [8]. The analysis showed that most of SNPs which related positively to salt tolerance indicators (RFW, RSL, and RWC) were on chromosome 7. Moreover, most of SNPs related negatively to salt tolerance indicators (relative electric conductivity and relative methylene dioxyamphetamine content,) were on chromosome 3 [8]. In the mentioned SNP-rich region, the authors identified candidate genes possibly associated with salt tolerance in *G. arboreum*, suggesting that this information could provide essential knowledge which would be very useful to the breeder in order to produce new salt tolerant cotton cultivars [8].

## 2. Transgenic Strategy

The use of transgenic plants to study the response to salinity is a strategy widely addressed in numerous research groups for many years. The homolog of *More Axillary Branches 2* (*MAX2*) encodes for a key component in the strigolactones (SL) signalling pathway. The overexpression of *MAX2* from *Sapium sebiferum* (*SsMAX2*) in Arabidopsis plants significantly promoted resistance to different abiotic stresses (drought, osmotic, and salinity) [9]. The authors showed that the protein MAX2 potentially influences chlorophyll metabolism, anthocyanin biosynthesis, soluble sugars, and proline accumulation. The physiological and biochemical analyses demonstrated that SsMAX2 plays a pivotal role in the regulation of redox homeostasis via the regulation of antioxidative enzymes. In this sense, a potential interaction between SL and abscisic acid (ABA) in the adaptation to abiotic was suggested [9].

Rice nucleolin protein (OsNUC1), consisting of two isoforms, OsNUC1-L and OsNUC1-S, is a multifunctional protein involved in salt-stress tolerance. The overexpression of *OsNUC1-S* gene improved rice productivity under saline conditions, which correlated with a better behaviour of gas exchange parameters (net photosynthesis, stomatal conductance, and transpiration rate) and higher carotenoids contents [10].

Rho-like GTPases (ROPs) from plants are a subfamily of small GTP-binding proteins crucial for plant survival when subjected to abiotic stress. Miao et al. [11] described a novel *ROP* gene from banana (*MaROP5g*) whose overexpression increased the tolerance to salt stress in transgenic *Arabidopsis thaliana* plants. This response was related to minor injury in the plasmatic membrane, as has been shown by reduced lipid peroxidation and electrolyte leakage values. It was also related to an increase in the cytosolic K^+^/Na^+^ ratio and the Ca^2+^ concentration. In addition, the *MaROP5g* overexpression up-regulated the salt overly sensitive (SOS)-pathway genes and several genes encoding calcium-signalling pathway proteins, including calcineurin B-like (CBL) proteins, CBL-interacting protein kinases (CIPKs), and calcium-dependent protein kinases (CDPKs) [12].

Bernal-Vicente et al. [13] studied the response to salt stress of a transgenic plum line (J8-1) harbouring four copies of the cytosolic ascorbate peroxidase gene (*cytapx*) from pea. The authors reported that this plum line was more tolerant to salinity stress in terms of plant growth, chlorophyll contents, chlorophyll fluorescence parameters, and root water contents. In addition, they proposed a connection between the salicylic acid and cyanogenic glucoside (CNglcs) biosynthetic pathways under salt-stress conditions [13].

The overexpression of the cucumber *TGase* (transglutaminase) gene from *Cucumis sativus* L. (*CsTGase*) in tobacco effectively ameliorated salt-induced photoinhibition by increasing the levels of polyamines (PAs) in the chloroplast as well as gas exchange and chlorophyll fluorescence parameters, along with greater abundance of D1 and D2 proteins under saline conditions [14]. In addition, *TGase* overexpression resulted in chloroplasts that showed more quantity and size of grana compared with wild type plants, suggesting a role of *TGase* in the chloroplast development. Thus, overexpression of *TGase* may be an effective strategy for enhancing resistance to salt stress in crops especially sensitive for agronomic production [14].

## 3. Physiological and Biochemical Mechanisms

Zhang et al. [15] presented a review about the physiological and molecular responses of *Populus* sp. to salinity. Poplars are used as a model species to study physiological and molecular responses of trees to NaCl stress, taken into account that salinity is one of the limiting factors of afforestation programs. The authors compared the response of salt-tolerant and salt-sensitive *Populus* species in terms of salinity injury (plant growth, photosynthesis) and primary salt-tolerance mechanisms (ion homeostasis, accumulation of soluble osmolytes), with reactive oxygen species (ROS) and reactive nitrogen species (RNS) metabolism and signaling networks induced by salinity, and they identified candidate genes for improving salt tolerance in the *Populus* sp.

## 4. Biostimulants and Salt-Stress Response

### 4.1. Biostimulants

The use of biostimulants is another strategy addressed to overcome the negative effects of salinity. Zhan et al. [16] presented an excellent review about the effect of melatonin in the plant response to salinity. They described the effects of exogenous melatonin in the modulation of the expression of genes involved in melatonin metabolism, the increase of the transcript levels of different stress-responsive genes, and transcription factors involved in the ROS scavenging and of the genes responsible for the maintenance of ion homeostasis. Melatonin also regulates hormone metabolism by up-regulation of gibberellic acid (GA) biosynthesis and abscisic acid (ABA) catabolism genes [16]. Finally, the authors described the identification of a plant melatonin receptor in Arabidopsis [17]. These finding opens new perspectives of research on the role of melatonin in response to abiotic stresses in general, and to salinity in particular.

Related to the previous revision, Zhao et al. [18] described that the treatment of *Brassica napus* L. seedlings with melatonin and NO-releasing compounds such as sodium nitroprusside (SNP), diethylamine NONOate (NONOate), and S-nitrosoglutathione (GSNO) produced synergistic effects that counteracted the seedling growth inhibition induced by NaCl exposure. At the same time, such treatments re-established the redox and ion homeostasis, by decreasing the ROS and lipid peroxidation accumulation as well as the Na^+^/K^+^ ratio. The addition of PTIO (a NO-scavenger) impaired the coupled response of melatonin and SNP, suggesting that NO is required to potentiate the effects of melatonin in protecting plants from salt stress [18]. Chen et al. (2018) [19] studied the salt-stress response of *Apocynum venetum* L plants, used in traditional Chinese medicine. The authors studied the changes in photosynthetic pigments, osmolytes, lipid peroxidation, some antioxidant enzymes, and ascorbic acid. By using UFLC-QTRAP-MS/MS technology a total of 43 bioactive constituents, including amino acids, nucleosides, organic acids, and flavonoids were successfully identified to change in response to salt stress. They applied a multivariate statistical analysis to evaluate the quality of *Apocynum venetum* L plants grown under saline conditions [19].

### 4.2. Plant Hormones

The role of root ABA (including ABA translocation from root to leaf) in the protection of photosystems and photosynthesis against salt stress was studied in Jerusalem artichoke [20]. In this study, the pretreatment of Jerusalem artichoke plants with sodium tungstate (a specific ABA synthesis inhibitor) followed by exposure to salt stress (150 mM NaCl) induced a drastic overaccumulation of Na^+^ in leaves. Moreover, a decline in net photosynthesis, ØPSII (actual photochemical efficiency of photosystem II) and Fv/Fm (the maximal photosystem II (PSII) quantum yield) was produced, indicating photoinhibition of PSII, along with the establishment of an oxidative stress due to an increase in H_2_O_2_ and lipid peroxidation levels. These results suggest that root ABA can participate in protecting PSII against photoinhibition in Jerusalem artichoke under salt stress, likely via a reduction of Na^+^ toxicity. In that regard, it has been reported that Na^+^ can irreversibly inactivate PSII and PSI by inducing secondary oxidative injury or through direct damage on photosynthetic proteins [21,22]. This finding was corroborated by immunoblotting analysis, where a decline in the PSII reaction center protein (PsbA) abundance was observed [20].

### 4.3. Protein Kinases, ROS, and Ion Homeostasis

Szymanska et al. [23] and Zhang et al. [24] described the involvement of different protein kinases families in the regulation of plant adaptation to salt stress [23,24]. Specifically, Szymanska et al. [23] showed that the SNF1-related protein kinases (SnRK2.4 and SnRK2.10) have a role in the modulation of ROS homeostasis in response to salinity by regulating the expression of several genes related to ROS generation and scavenging in Arabidopsis.

Zhang et al. [24] described the importance of CDPKs (Ca^2+^-dependent protein kinases) in the adaptation of Arabidopsis to salt stress. In that regard, they reported that the *CPK12*-RNA interference (RNAi) mutant was more sensitive to salinity than the wild-type plants in terms of seedling growth. This response seemed to be related to the accumulation of phytotoxic ions in the roots as well as the overgeneration of H_2_O_2_ in the *CPK12*-RNAi mutants [24].

Regarding the effect of salt stress in ion homeostasis, Ali et al. [25] provided a brief overview of the role of the high-affinity potassium-type transporter 1 (HKT1) and their importance in different plant species under salt stress. HKT1-type transporters play a crucial role in Na^+^ homeostasis, being of pivotal importance to maintain an optimal K^+^/Na^+^ balance in the cytoplasm in response to salt stress for plant survival. The authors described the role of HKT1-type transporters and their functional differences in glycophytes and halophytes [25].

Luo et al. [26] showed that Arabidopsis plants overexpressing a SKn-type dehydrin from *Capsicum annuum* L. (*CaDHN5*) resulted an increased tolerance to salt and osmotic stress, suggesting an important role for *CaDHN5* in response to the mentioned abiotic stresses [26]. In addition, using VIGS (virus-induced gene silencing) technique, the authors reported that knockdown of the *CaDHN5* gen suppressed the expression of manganese superoxide dismutase (*MnSOD*) and peroxidase (*POD*) genes in transformed pepper plants [26]. These changes caused a higher oxidative stress in the VIGS lines than in control pepper plants under NaCl or osmotic stress conditions, as observed by the data of some stress-oxidative parameters (superoxide accumulation, lipid peroxidation, and electrolyte leakage), chlorophyll levels, and the rate of water loss. The results demonstrated an important role for the *CaDHN5* gene in the tolerance of plants to salt and osmotic stresses as well as in the salt and osmotic stress signalling pathways [26]. The results also indicated that *CaDHN5* positively regulates the expression of the *MnSOD* and *POD* genes, but also other stress-related genes, including *AtSOD1* (encoding a H^+^/Na^+^ plasma membrane antiporter), *AtDREB2A* (a transcription factor in the ABA signalling pathway), and *AtRSA1* and *AtRITF1* genes that regulate the transcription of several ROS scavenging-related genes and the *AtSOS1* gene [26].

## 5. Proteomic Approach

The isobaric tags for relative and absolute quantitation (iTRAQ)-based proteomic technique was used to identify the differentially-expressed proteins in leaves of two rice genotypes that differ in their tolerance to salt stress [27]. The iTRAQ protein profiling identified in both rice genotypes revealed that the differentially-expressed proteins were mainly involved in the regulation of salt-stress responses, in oxidation-reduction responses, in photosynthesis, and in carbohydrate metabolism. Regarding their subcellular localization, most of them were predicted to localize in cytoplasm and chloroplasts (67.2% of the total up-regulated proteins) [27].

## 6. Conclusions and Outlook

Salinity is one of the major factors that limits geographical distribution of plants and adversely affects crop productivity and quality worldwide. Salinization affects about 30% of the irrigated land of the world, increasing this area approximately 1–2% per year due to salt-affected land surfaces (FAO, 2014). In Europe, about 3 million hectares of the land are affected by salinization. Unfortunately, this situation will worsen in a context of climate change, where there will be an overall increase in temperature and a decrease in average annual rainfall worldwide.

Although an important part of the studies on response to salinity are carried out with Arabidopsis plants, nowadays the use of other species with agronomic interest is also remarkable, including woody plants.

Studies on salinity tolerance have focused on different points of view: agronomic, physiological, biochemical, and molecular. However, in recent years, the number of works that address tolerance to salinity from a molecular point of view has increased considerably, in order to search for candidate genes that may be useful to the search for resistant genotypes. The identification of the candidate genes would provide valuable information about the molecular and genetic mechanisms involved in the salt tolerance response, and it would also supply important resources to the breeding programs in order to look for salt tolerance in crop plants. Therefore, obtaining salt-tolerant species is one of the goals for breeders, and probably, the use of transformed plants could improve the salt response in crop plants. In this way, transformed plants with enhanced antioxidant defenses have been obtained in different laboratories, and, in most cases, these plants displayed an improved salt-tolerance response. The overexpression of certain proteins can afford protection against salt stress in plants. In this Special Issue, the author shows that the overexpression of certain transgenes improved the response to salinity in plants in terms of photosynthesis rate, improved the gas exchange parameters, and increased photosynthetic pigments, antioxidant mechanisms, and accumulation of anthocyanins, as well as improved ion homeostasis responses, up-regulation of ABA biosynthesis genes, and plant hormone signaling. However, the use of transgenic plants for agricultural purposes still has a high level of rejection by consumers, for example in the European Union, motivated by its agricultural policy.

Another feasible strategy to mitigate salinity impacts on crop production would be breeding salt tolerant cultivars for the production of new varieties which can thrive in more extreme environmental conditions. In this sense, crop wild relatives may contain genes of potential value for plant salinity tolerance. Despite the vast pool of resources that exists, much of the crop germplasm richness found in gene banks is underutilized.

In addition, cultivation of halophytic plants at the same time or prior to the cultivation of crop plants (intercropping) would allow the desalination of the soil favoring crop yield and/or, alternatively, the use of saline irrigation water. Complementarily, the use of biostimulants, such as antioxidant compounds, melatonin, plant hormones or NO-releasing compounds can improve the response of plants to salinity.

The proteomic approach to study the response to salt stress can provide relevant information in order to know the physiological and biochemical processes affected by salinity. This information can also support the breeding programs to attempt selection of salt-tolerant plants.

Finally, the development of plant metabolomics techniques can supply relevant information about the effect of salt stress on cell metabolism. In addition, these techniques may allow for the identification of new metabolites that can be used as markers to better understand the salt tolerance response and help breeders select new tolerant species.
